# Parkinson’s disease-related DJ-1 functions in thiol quality control against aldehyde attack *in vitro*

**DOI:** 10.1038/s41598-017-13146-0

**Published:** 2017-10-09

**Authors:** Noriyuki Matsuda, Mayumi Kimura, Bruno Barros Queliconi, Waka Kojima, Masaki Mishima, Kenji Takagi, Fumika Koyano, Koji Yamano, Tsunehiro Mizushima, Yutaka Ito, Keiji Tanaka

**Affiliations:** 1 Ubiquitin Project, Tokyo Metropolitan Institute of Medical Science, 2-1-6 Kamikitazawa, Setagaya, Tokyo, 156-8506 Japan; 2 JST, PRESTO, 4-1-8 Honcho, Kawaguchi, Saitama, 332-0012 Japan; 3 Laboratory of Protein Metabolism, Tokyo Metropolitan Institute of Medical Science, 2-1-6 Kamikitazawa, Setagaya, Tokyo, 156-8506 Japan; 4 Department of Computational Biology and Medical Sciences, Graduate School of Frontier Sciences, The University of Tokyo, 5-1-5 Kashiwanoha, Kashiwa, Chiba, 277-8561 Japan; 5 Graduate School of Science and Engineering, Tokyo Metropolitan University, 1-1 Minamiosawa, Hachioji, 192-0397 Japan; 6 Picobiology Institute, Graduate School of Life Science, University of Hyogo, 3-2-1 Kouto, Kamighori, Ako, Hyogo, 678-1297 Japan

## Abstract

*DJ-1* (also known as *PARK7*) has been identified as a causal gene for hereditary recessive Parkinson’s disease (PD). Consequently, the full elucidation of DJ-1 function will help decipher the molecular mechanisms underlying PD pathogenesis. However, because various, and sometimes inconsistent, roles for DJ-1 have been reported, the molecular function of DJ-1 remains controversial. Recently, a number of papers have suggested that DJ-1 family proteins are involved in aldehyde detoxification. We found that DJ-1 indeed converts methylglyoxal (pyruvaldehyde)-adducted glutathione (GSH) to intact GSH and lactate. Based on evidence that DJ-1 functions in mitochondrial homeostasis, we focused on the possibility that DJ-1 protects co-enzyme A (CoA) and its precursor in the CoA synthetic pathway from aldehyde attack. Here, we show that intact CoA and β-alanine, an intermediate in CoA synthesis, are recovered from methylglyoxal-adducts by recombinant DJ-1 purified from *E. coli*. In this process, methylglyoxal is converted to L-lactate rather than the D-lactate produced by a conventional glyoxalase. PD-related pathogenic mutations of DJ-1 (L10P, M26I, A104T, D149A, and L166P) impair or abolish detoxification activity, suggesting a pathological significance. We infer that a key to understanding the biological function of DJ-1 resides in its methylglyoxal-adduct hydrolase activity, which protects low-molecular thiols, including CoA, from aldehydes.

## Introduction

Genetic studies on the hereditary forms of Parkinson’s disease (PD) have identified multiple genes relevant to disease pathogenesis. *DJ-1* (also known as *PARK7*) has been identified as a causal gene for hereditary recessive Parkinsonism^1^. Since loss-of-function mutations in *DJ-1* lead to familial Parkinsonism, DJ-1 usually counteracts a predisposition for the disease. Consequently, a better understanding of DJ-1 function will help decipher the molecular mechanisms underlying PD pathogenesis.

DJ-1 is a relatively small (189 amino acids; <20 kDa) multifunctional protein. Since first identified as an oncogene^2^, DJ-1 function has been the focus of several hundred studies, many of which have revealed the pleiotropic nature of the protein. For example, DJ-1 has been proposed to function as a regulator of the 20S proteasome^3–5^, a chaperone for alpha-synuclein^6^, a regulator of the androgen receptor^7^, a redox-sensitive esterase^8^, a peroxiredoxin-like peroxidase that scavenges H_2_O_2_^9^, a transcriptional regulator^10^, an RNA binding protein^11^, a regulator of tyrosine hydroxylase^12^, a protease^13^, a stabilizer of the antioxidant transcriptional regulator Nrf2^14^, a regulator of Bax^15^, and a transcriptional regulator of uncoupling (UCP) proteins^16^. Moreover, multiple lines of evidence, including genetic studies in model organisms, have suggested DJ-1 dysfunction sensitizes cells to oxidative stress and causes mitochondrial dysfunction^3,17–26^. However, because diverse, and often inconsistent, functions have been reported (as listed above), the research community has yet to reach a consensus on the molecular function of DJ-1.

A unique characteristic that clearly distinguishes DJ-1 from other Parkinson’s disease-relevant proteins such as PINK1, Parkin, and LRRK2 is its broad evolutionary conservation, which extends to the prokaryotic kingdom. DJ-1 belongs to the well-conserved PfpI/Hsp31/DJ-1 superfamily of proteins that are characterized by a conserved cysteine in a nucleophile elbow structure that resembles the catalytic cysteine in cysteine proteases^27–30^. Interestingly, this family of proteins is prevalent in many prokaryote species from Gram-negative, facultative anaerobic bacteria such as *Escherichia coli* (*E. coli*) to Gram-positive, low-GC firmicutes bacteria such as *Bacillus subtilis* (*B. subtilis*). The prokaryotic DJ-1 homolog YajL exhibits up to 38% pairwise identity (50% similarity) with the DJ-1 amino acid sequence, strongly suggesting that DJ-1 plays a well-conserved role in both prokaryotes and eukaryotic multicellular organisms.

Prokaryotic DJ-1 homologs can provide many insights into the intrinsic function of DJ-1. YraA, the *B. subtilis* homolog of DJ-1, comprises an operon with formaldehyde dehydrogenase (AdhA). In addition, both are simultaneously expressed as an *adhA-yraA* bicistronic transcript when cells are treated with electrophilic carbonyls such as formaldehyde and methylglyoxal (MG)^31^. As a result, YraA has been suggested to function in aldehyde detoxification. The *E. coli* Hsp31/HchA protein, which has significant sequence similarity with DJ-1, functions as a glyoxalase III that directly converts methylglyoxal to lactate^32^. Since methylglyoxal (also referred to as pyruvaldehyde) is an aldehyde, the glyoxalase III activity of HchA/Hsp31 implicates DJ-1 family proteins in aldehyde detoxification. Furthermore, *Candida albicans* Hsp31/HchA and human DJ-1 have been reported to exhibit glyoxalase III activity^33,34^. Moreover, disruption of these DJ-1 homologs (YraA and Hsp31/HchA) sensitizes prokaryotic cells to exogenous aldehyde^31,32^.

Here, we show that DJ-1 converts methylglyoxal-adducted small molecular thiols, including glutathione (GSH), cysteine, and coenzyme A (CoA), to intact thiol and L-lactate. We propose that DJ-1 functions as a hydrolase of aldehyde-adducts for homeostatic control of thiol quality, which protects low-molecular-weight thiols from aldehyde attack.

## Results

### DJ-1 converts glutathione-adducted methylglyoxal to L-lactate

The molecular mechanism of methylglyoxal (CH_3_COCHO) detoxification in terms of the glyoxalase I (GloI)/ glyoxalase II (GloII)-mediated “two-step detoxification system” is well characterized. In this pathway, the hemithioacetal [CH_3_COCH(OH)-S-glutathione] formed from spontaneous reaction of methylglyoxal and intracellular glutathione (GSH) undergoes isomerization by GloI to yield S-D-lactoyl-glutathione [CH_3_CH(OH)CO-S-glutathione]. The thioester bond is then hydrolysed by glyoxalase ІІ to generate D-lactate and GSH. Collectively, the sequential actions of GloI and GloII enzymes convert methylglyoxal to non-reactive D-lactate^35,36^.

**Figure 1:**
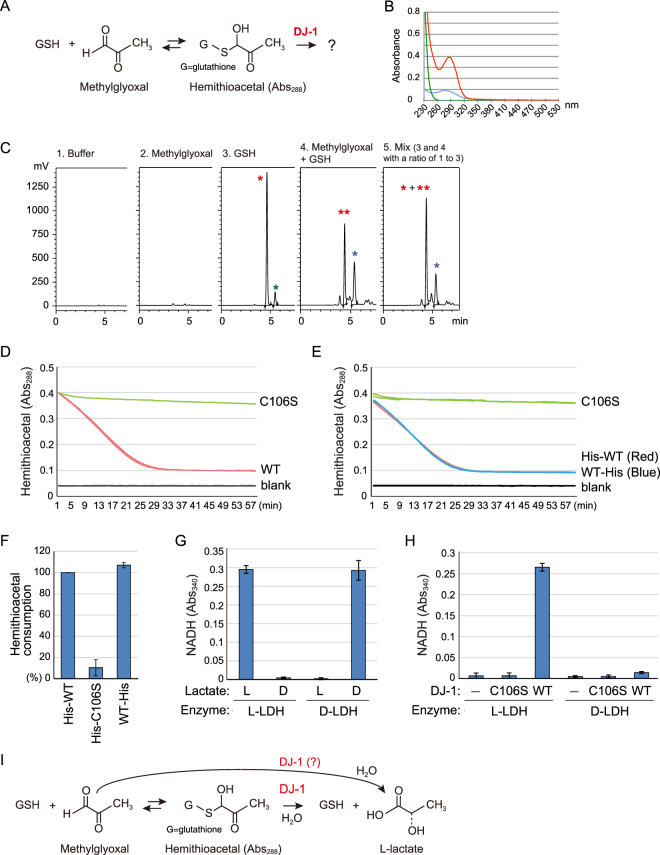
(**A**) Reaction mechanism for hemithioacetal formation. (**B**) Glutathione (GSH) and methylglyoxal react to produce hemithioacetal with a characteristic absorbance at 288 nm. Green, blue, and red lines indicate spectra for GSH, methylglyoxal, and a reacted mixture. (**C**) Confirmation of hemithioacetal formation by HPLC analysis. New peak (blue asterisk) derived from methylglyoxal-conjugated GSH (hemithioacetal) was observed. (**D**) The Abs_288_ of GSH-derived hemithioacetal decreased when incubated with wild type (WT) DJ-1 (red) but not the C106S mutant (green). The black line indicates a blank control without DJ-1. (**E**) The time course for hemithioacetal consumption by carboxyl-terminal His_6_-tagged DJ-1 (blue) and amino-terminal His_6_-tagged DJ-1 (red) are indistinguishable. (**F**) Degree of hemithioacetal consumption. Values are based on the linear portion of the degradation curves shown in (**D**) and (**E**) relative to amino-terminal His_6_-tagged WT DJ-1, which is defined as 100% consumption. (**G**) Two types of lactate dehydrogenase (L-LDH and D-LDH) differentiate between L-lactate and D-lactate. (**H**) When WT DJ-1 was incubated with GSH and methylglyoxal, L-lactate but not D-lactate was specifically produced. In (**F**), (**G**), and (**H**), bars represent the mean ± SD of three experiments. (**I**) Proposed reaction mechanism for DJ-1 conversion of methylglyoxal-conjugated GSH.

Although DJ-1 directly converts methylglyoxal to lactate^34^ and methylglyoxal-conjugated N-acetyl-cysteine to intact cysteine and lactate^37^, its functional similarity with GloI and GloII in generating lactate from methylglyoxal-adducted GSH (Fig. 1A) remains undefined.

To insure a robust assay system for monitoring hemithioacetal formation, we reacted 7.5 mM methylglyoxal and 7.5 mM GSH for 15 min, and then examined absorbance at 288 nm (Abs_288_) for the characteristic hemithioacetal peak^38^ (Fig. 1A,B). The increase in Abs_288_ was only observed when methylglyoxal and GSH were reacted (Fig. 1B, red line). Using the same concentrations (7.5 mM) of methylglyoxal (Fig. 1B, blue line) or GSH (Fig. 1B, green line) in separate reactions resulted in either weak or no absorbance respectively. We also utilized an HPLC method to monitor hemithioacetal formation (Fig. 1C). Using conditions for detecting GSH, a clear peak (t = 4.5 min, red asterisk in panel 3) was observed with GSH alone, whereas no peak was observed with methylglyoxal alone (panel 2). However, when methylglyoxal and GSH were mixed, a new chromatographic peak (t = 5.5 min, blue asterisk in panel 4) corresponding to the methylglyoxal-adducted GSH hemithioacetal was generated. The first peak (double red asterisks) in panel 4 is derived from GSH, because this peak merged completely with the main peak in panel 3 that corresponded to GSH alone (panel 5). The minor peak in panel 3 (green) is not methylglyoxal-adducted GSH (blue) because the ∆peaks value (0.837) is inconsistent with the ∆peaks values (0.970 and 0.972, respectively) in panels 4 and 5, and is likely derived from an impurity in GSH or the HPLC column.

To assess the role of DJ-1 on hemithioacetal formation, recombinant wild-type (WT) DJ-1 and a mutant in which the putative catalytic cysteine was serine-substituted (*i.e*. C106S) were purified from *E. coli* using an amino-terminal His_6_-tag. The quality of the purifications was confirmed by CBB with each protein resolving as a single band (Fig. S1). Reaction mixtures of methylglyoxal and GSH were then incubated with WT DJ-1 or the C106S mutant. We observed a decrease in Abs_288_ in the presence of WT DJ-1 but not in the presence of the C106S mutant (Fig. 1D,F). DJ-1 thus degrades hemithioacetal *in vitro* depending on availability of the putative Cys106 catalytic center. To examine potential confounding effects of the amino-terminal His_6_-tag, we generated carboxyl-terminal His_6_-tagged versions of WT DJ-1 and the C106S mutant and found that hemithioacetal was similarly consumed (Fig. 1E,F). Next, we examined whether lactate, the expected final product, is produced. Similar to the GloI/GloII system^35,36^, DJ-1 has been reported to mediate the conversion of methylglyoxal to D-lactate^37,39^. However, a separate study suggested that DJ-1 generates L-lactate^40^. To better clarify this detail, we used a classical lactate-monitoring assay in which lactate dehydrogenase (LDH) converts lactate to pyruvate by coupling the reaction to production of NADH from NAD. The unique Abs_340_ of the resulting NADH was then monitored spectrophotometrically^41^. Using enantiomer-specific forms of LDH (D-LDH and L-LDH), we were able to differentiate the two enantiomers (Fig. 1G). L-lactate production was observed clearly when WT DJ-1 was incubated with methylglyoxal and glutathione-derived hemithioacetal, whereas D-lactate was rarely detected under the same conditions (Fig. 1H, column 3 and 6). The C106S mutant produced neither type of lactate (Fig. 1H, column 2 and 5). We thus conclude that DJ-1 converts GSH-adducted methylglyoxal to L-lactate (Fig. 1I).

### Prokaryotic DJ-1 orthologue implicated in the coenzyme A synthetic pathway

**Figure 2:**
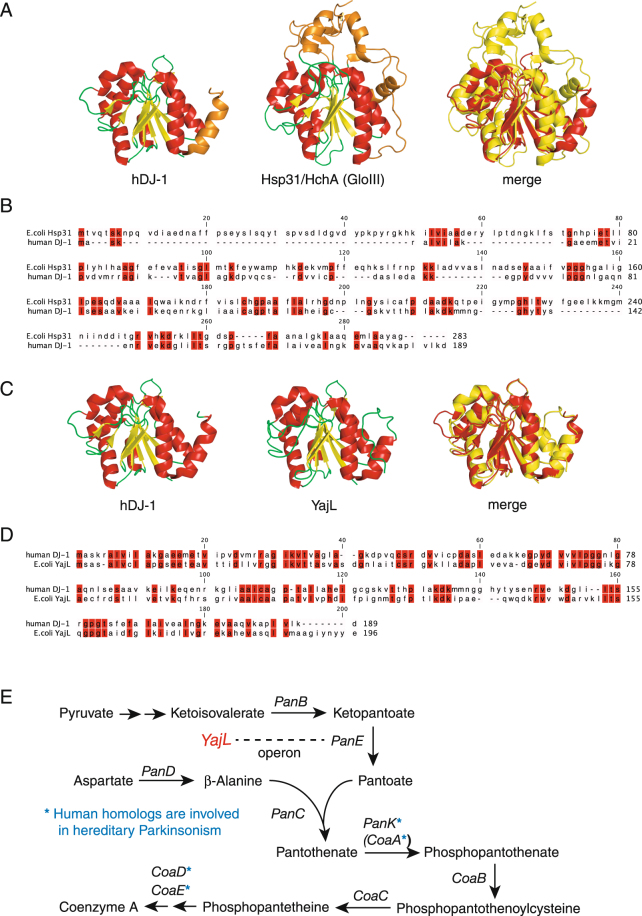
(**A**) Structural comparison of human DJ-1 (hDJ-1, left) and *E. coli* Hsp31/HchA (middle), which has glyoxalase III (GloIII) activity. Non-conserved elements between hDJ-1 and Hsp31/HchA are indicated in orange. The merged figure (right) shows superposition of the hDJ-1 (red) and Hsp31/HchA (yellow) structures. (**B**) Sequence alignment of hDJ-1 and Hsp31/HchA. Identical amino acids are highlighted in red. (**C**) and (**D**) Structure and sequence comparison of human DJ-1 with *E. coli* YajL as in (**A**) and (**B**). (**E**) Coenzyme A (CoA) biosynthetic pathway in *E. coli*. *PanE* comprises an operon with *YajL*, an *E. coli* orthologue of DJ-1.

Even if DJ-1 degrades “methylglyoxal-adducted GSH” to L-lactate and GSH *in vivo*, the GloI/II system can detoxify it, implying that this DJ-1 function is redundant under physiological conditions. We thus speculated that additional genuine targets of DJ-1 should exist. DJ-1 has been proposed to function in mitochondrial homeostasis^3,17–26^. When considered within this context, the relationship between thiols like coenzyme A (CoA) and mitochondrial function becomes apparent as the CoA-derivatives acetyl-CoA and succinyl-CoA are essential for mitochondrial production of ATP^42^. Moreover, comparative analyses with the prokaryotic DJ-1 homolog likewise implicate a role in the CoA-biosynthetic pathway. Although *E. coli* deglycase III (Hsp31/HchA) has significant sequence similarity with human DJ-1^32^, several domains of Hsp31/HchA and DJ-1 are not reciprocally conserved (Fig. 2A, shown in orange). Hsp31/HchA has 30% amino acid identity to DJ-1 (Fig. 2B); however, the *E. coli* genome encodes other homologous protein, YajL, that has higher sequence similarity with DJ-1. Superposition of their structures reveals that YajL and DJ-1 have almost identical backbone trajectories with an Cα RMSD value of 2.01 Å (Fig. 2C). At the amino acid sequence level, YajL is 38% identical with 50% similarity to DJ-1, confirming that YajL is the closest *E. coli* homolog of human DJ-1 (Fig. 2D)^43^. Although structural and sequence comparisons suggest that Hsp31/HchA is homologous to DJ-1, the *E. coli* orthologue of DJ-1 is YajL.

A search of the prokaryotic operon databases ODB3 (http://operondb.jp/)^44,45^ and DOOR (http://csbl.bmb.uga.edu/DOOR/)^46,47^, revealed that *YajL* comprises an operon with 2-dehydropantoate 2-reductase *PanE*. PanE converts ketopantoate to pantoate, and the resultant pantoate reacts with β-alanine to produce pantothenate, an essential CoA intermediate product (Fig. 2E, also see Discussion).

We hypothesized that DJ-1 protects CoA or CoA precursors from aldehyde conjugation given that: 1) DJ-1 likely functions in mitochondrial homeostasis, 2) prokaryotic DJ-1 family proteins such as YraA and Hsp31/HchA are involved in aldehyde detoxification, and 3) *YajL*, the prokaryotic *DJ-1* orthologue, comprises an operon with *PanE* that functions in the CoA biosynthetic pathway. We thus examined whether DJ-1 restores methylglyoxal-adducted CoA (the final thiol product) and β-alanine (an intermediate amine).

### Intact CoA and β-alanine are recovered from methylglyoxal-adducts by DJ-1

**Figure 3:**
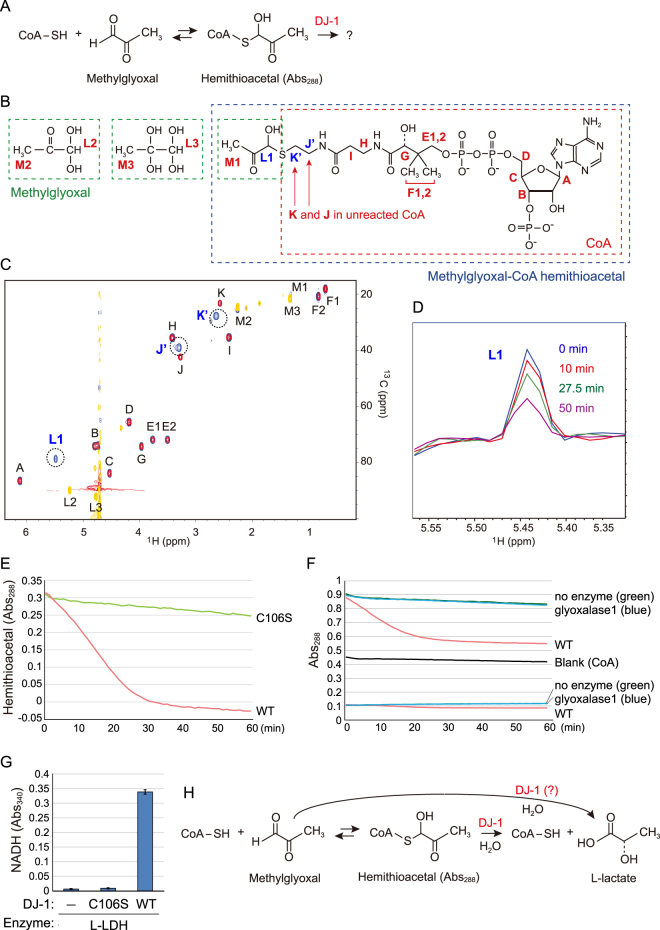
(**A**) Reaction of coenzyme A (CoA) and methylglyoxal to produce hemithioacetal. (**B**) Possible molecular species present in the reaction. A - M indicate positions of the chemical shift assignments. (**C**) HSQC spectra of reacted methylglyoxal and CoA (blue) are superimposed with spectra for methylglyoxal alone (yellow) and CoA alone (red). (**D**) The L1 peak was reduced when incubated with DJ-1. (**E**) The Abs_288_ of CoA-derived hemithioacetal decreased when incubated with wild type (WT) DJ-1 but not the C106S mutant. The Abs_288_ of the reaction lacking DJ-1 was subtracted as a blank control. (**F**) Abs_288_ of CoA-derived hemithioacetal (upper lines) or methylglyoxal (lower lines) when incubated with DJ-1 or yeast glyoxalase I. (**G**) L-lactate was produced only when WT DJ-1 was incubated with CoA and methylglyoxal. No lactate was produced in the presence of the C106S mutant. Bars represent the mean ± SD. (**H**) Proposed reaction mechanism for DJ-1-mediated conversion of methylglyoxal-conjugated CoA.

To obtain unambiguous evidence for methylglyoxal-adduct CoA, we used the two-dimensional NMR methods heteronuclear single-quantum correlation (HSQC) spectroscopy and heteronuclear multiple bond correlation (HMBC) spectroscopy. Methylglyoxal and CoA were initially reacted for 15 min prior to the experimental assay to generate hemithioacetal [CH_3_COCH(OH)-S-CoA] (Fig. 3A). Although methylglyoxal contains a single methyl group (CH_3_ moiety) and a methine group (C-H moiety), the molecule can assume both monohydrate and dihydrate forms in aqueous solution (Fig. 3B, left) and thus yield two signals (M2/M3 and L2/L3) in NMR experiments (Fig. 3C, yellow), as reported previously^48^. Superimposition of HSQC spectra for unreacted CoA (red) and unreacted methylglyoxal (yellow) onto the spectrum of the methylglyoxal and CoA reaction mixture (blue) is shown in Fig. 3C. By comparing the unreacted reagent signals with those from the mixture, we identified three product-derived signals: K’ (ω2 = 2.64, ω1 = 27.8 ppm), J’ (ω2 = 3.30, ω1 = 39.0 ppm), and L1 (ω2 = 5.48, ω1 = 78.7ppm) (Fig. 3C, encircled).

We next analyzed these signals by tracing the connectives obtained by HMBC. The ^13^C chemical shift of the cross peak in ω2 = 5.48 ppm, that corresponds to L1, is 27.8 ppm. This clearly matches the ^13^C chemical shift of K’, and conversely, the ^13^C chemical shift (78.7 ppm) of the cross peak in ω2 = 2.64 ppm (correspond to K’) matches that of L1 (Fig. S2). This indicates that L1 and K’ are adjacent. Likewise, based on the K’ and J’ connectives derived from the HMBC cross peaks, we concluded that K’ and J’ are adjacent. Further, ^13^C and ^1^H chemical shifts of L1 are consistent with L1 as the CH moiety, whereas the chemical shifts of J’ and K’ are consistent as the CH_2_ moiety. Taken together, L1, J’, and K’ were unambiguously assigned to the reaction product (methylglyoxal-adduct CoA) as shown in Fig. 3B.

Since L1 is the signal derived from the reaction product and is well dispersed even in the 1D slice of the HSQC spectrum, we sought to determine if DJ-1 diminishes the L1 signal in 1D-HSQC. This experimental approach offers high sensitivity and enhanced performance under water-suppression conditions. Of note, we avoided the use of normal ^1^H 1D NMR since its water-suppression often causes confounding base line distortions that interfere with signal intensity interpretation. In the experimental analysis, the signal intensity of L1 decreased within an hour of DJ-1 addition (Fig. 3D), whereas the signal intensity remained unchanged in the absence of DJ-1 (Fig. S3). Moreover, we observed an increase in lactate-derived signals. Lactate contains a single methyl group (CH_3_ moiety, M4 in Fig. S4A) and a methine group (C-H moiety, L4 in Fig. S4A) that provide 1.2 and 4 ppm signals in 1D-HSQC, respectively. We observed that these two signals (1.2 ppm and 4 ppm) increased over time in the presence of DJ-1, indicating new lactate production (Fig. S4B). These 1D-HSQC results confirm that DJ-1 generates lactate from methylglyoxal-adducted CoA.

The degradation of hemithioacetal by WT DJ-1 or the C106S mutant was also monitored by a change in Abs_288_ as in Fig. 1. The hemithioacetal-derived Abs_288_ decreased in the presence of WT DJ-1 but did not in the presence of the C106S mutant (Fig. 3E), indicating consumption of methylglyoxal-adducted CoA. When the activity of DJ-1 on methylglyoxal itself was monitored, the methylglyoxal-derived Abs288 signal (lower red line in Fig. 3F) had a much lower absolute value (Fig. 1B), but the reduction rate was less than methylglyoxal-adducted CoA (upper red line in Fig. 3F). The different kinetics of DJ-1 activity on methylglyoxal alone and methylglyoxal-adducted CoA suggests that DJ-1 directly functions on methylglyoxal-adducted CoA. Interestingly, when a classical methylglyoxal detoxification enzyme (yeast glyoxalase I) was reacted with methylglyoxal-adducted CoA, no hemithioacetal degradation was observed (Fig. 3F, blue lines)(see Discussion). Lastly, we monitored L-lactate under the aforementioned conditions. Using the lactate monitoring assay from Fig. 1G, we confirmed L-lactate was produced by WT DJ-1 in the presence of methylglyoxal- and CoA-reacted hemithioacetal (Fig. 3G and H).

**Figure 4:**
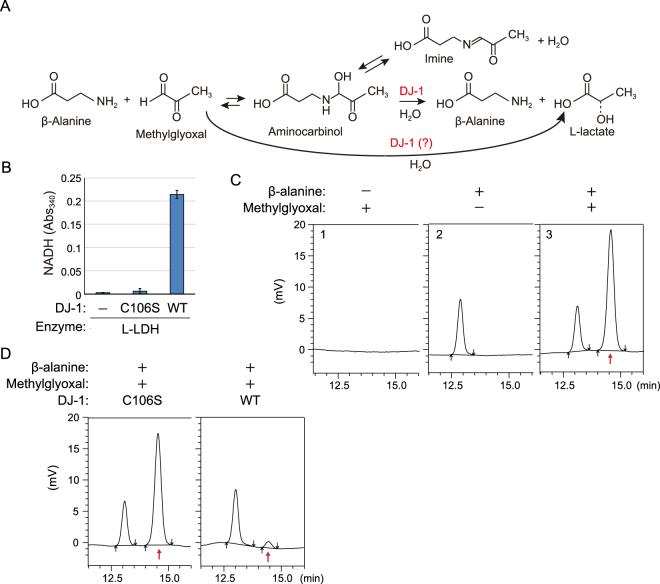
(**A**) Reaction mechanism for the generation of aminocarbinal and imine from β-alanine and methylglyoxal (upper reaction), and the proposed reaction mechanism for the role of DJ-1 in detoxification. (**B**) Production of L-lactate by WT DJ-1 from a mixture of pre-incubated β-alanine and methylglyoxal. Bars represent the mean ± SD. (**C**) HPLC confirmation of aminocarbinal formation. A new peak (t = 14.5 min, red arrow) derived from methylglyoxal-conjugated β-alanine was observed. (**D**) WT DJ-1, but not the C106S mutant, decreases methylglyoxal-conjugated β-alanine (red arrow) with a reduction in the reaction peak.

Similarly, we examined whether DJ-1 restores methylglyoxal-adducted β-alanine. When β-alanine reacts with methylglyoxal, aminocarbinal and imine are produced (Fig. 4A, upper part). Methylglyoxal and β-alanine were reacted prior to the start of the assay, incubated with WT DJ-1 or the C106S mutant, and L-lactate production was monitored. L-lactate was again produced when WT DJ-1 was incubated with methylglyoxal and β-alanine, whereas the C106S mutant had no effect (Fig. 4B). Previously (Figs 1 and 3), hemithioacetal production was confirmed spectrophotometrically (Abs_288_); however, the expected products in this reaction, aminocarbinal and imine, cannot be monitored spectrophotometrically. We thus needed to rule out the trivial possibility that the methylglyoxal did not react with β-alanine in the first place. We utilized HPLC to monitor a decrease in the methylglyoxal-adducted β-alanine. Using conditions for detecting β-alanine, no peaks were observed in the presence of methylglyoxal alone (Fig. 4C, panel 1), whereas β-alanine alone generated a single peak (panel 2). A separate chromatographic peak corresponding to the methylglyoxal-adducted β-alanine appeared after co-incubation (panel 3, red arrow). Further incubation with WT DJ-1, but not the C106S mutant, diminished the peak, directly demonstrating that DJ-1 decreases methylglyoxal-adducted β-alanine (Fig. 4D). Because DJ-1 converts the methylglyoxal-adduct to restore CoA and β-alanine *in vitro* (Figs 3 and 4), we inferred that DJ-1 plays a role in maintaining the integrity of the CoA synthetic pathway.

### Pathogenic DJ-1 mutations affect the methylglyoxal detoxification activity of DJ-1

**Figure 5:**
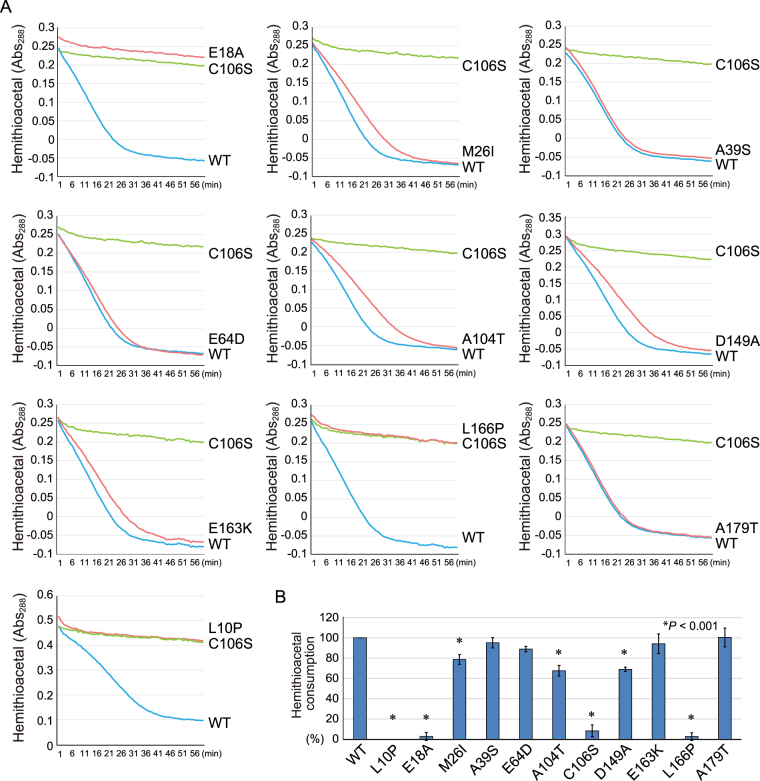
(**A**) Effect of various mutations on DJ-1 degradation of methylglyoxal-adducted CoA. The Abs_288_ profiles of CoA-derived hemithioacetal in the presence of various DJ-1 mutants over time are shown. The blue, green, and red lines correspond to WT, the C106S mutant, and pathogenic-based mutants, respectively. The Abs_288_ of the reaction lacking DJ-1 was subtracted as a blank control. (**B**) Degree of hemithioacetal consumption. Values are based on the linear portion of the degradation curves shown in (**A**) relative to WT DJ-1, which is defined as 100% consumption. Bars represent the mean ± SD (error bars) with statistical significance evaluated using one-way-ANOVA and Dunnett’s test.

*DJ-1* has been identified as a causal gene for hereditary recessive Parkinsonism^1^. To confirm the physiological and etiological importance of DJ-1 function, we selected various pathogenic mutations and examined their effects on enzymatic activity. Recombinant His_6_-tagged DJ-1 proteins with nine separate pathogenic mutations (L10P, M26I, A39S, E64D, A104T, D149A, E163K, L166P, and A179T) or two mutations of the putative catalytic amino acids (E18A and C106S) were purified from *E. coli* as single bands (Fig. S1). As before, hemithioacetal was generated from methylglyoxal and CoA prior to the start of the assay and Abs_288_ of the reaction in the presence of the DJ-1 mutants was monitored. The A39S and A179T mutants consumed methylglyoxal-adducted CoA in a manner similar to wild-type DJ-1 (Fig. 5A). In contrast, the L10P, M26I, A104T, D149A, and L166P mutants had compromised DJ-1 activity (Fig. 5A,B).

**Figure 6:**
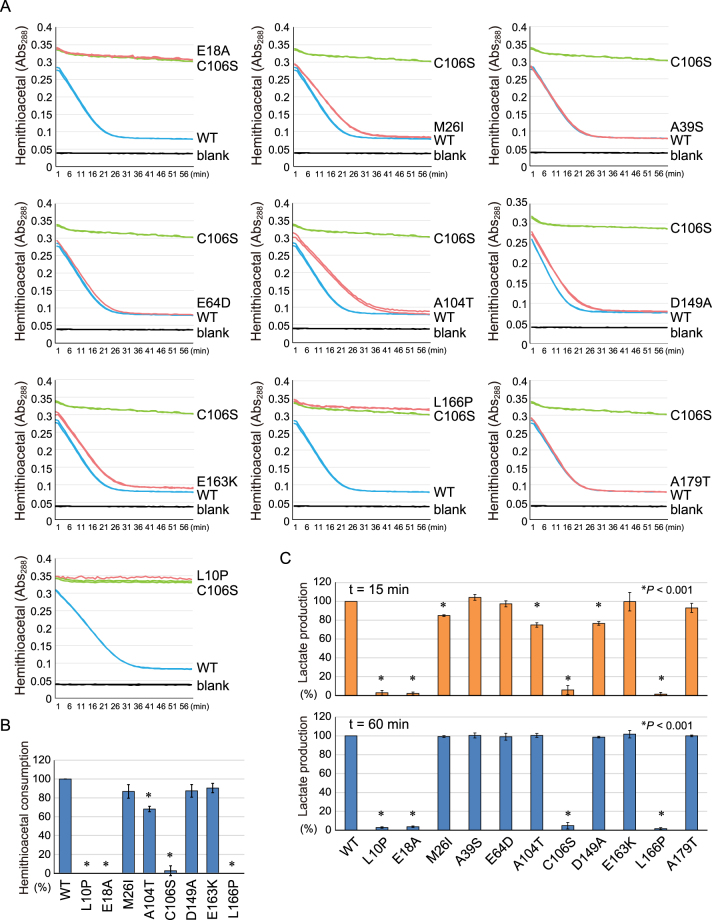
(**A**) A shift in the Abs_288_ of NAC-derived hemithioacetal when incubated with various DJ-1 mutants over time is shown. The blue, green, and red lines correspond to WT, the C106S mutant, and pathogenic-based mutants, respectively. The black line corresponds to the blank control without DJ-1. (**B**) Degree of hemithioacetal consumption. Values are based on the linear portion of the degradation curves shown in (**A**). (**C**) Lactate production of various DJ-1 mutants when incubated with NAC and methylglyoxal. In (**B**) and (**C**), values relative to WT DJ-1, which is defined as 100%, are shown. Bars represent the mean ± SD (error bars) with statistical significance evaluated using one-way-ANOVA and Dunnett’s test.

To examine the enzymatic activities of the various pathogenic DJ-1 mutants on additional substrates, we used the small molecular thiol, N-acetyl-cysteine^37^. N-acetyl-cysteine (NacCys) and methylglyoxal were reacted for 15 min prior to the start of the assay to generate hemithioacetal [CH3COCH(OH)-S-NacCys]. The reaction in the presence of WT DJ-1 or the various DJ-1 mutants was monitored at Abs_288_ as before. The A39S, E64D, and A179T DJ-1 mutants consumed methylglyoxal-adducted NacCys similar to WT DJ-1. In contrast, the reduction in hemithioacetal was slowed by the M26I, A104T, and D149A mutants and was completely inhibited by the L10P and L166P mutants (Fig. 6A). Statistical analysis confirmed the decrease in hemithioacetal consumption by L10P, A104T, and L166P pathogenic mutants (Fig. 6B). We then examined the effects of the DJ-1 mutants on lactate production. However, because the conditions (pH 9.0 and presence of hydrazine) required to monitor lactate production in real-time^41^ abolish DJ-1 activity, we hypothesized that differences in DJ-1 enzymatic activity (e.g. the A104T mutant) would be concealed if lactate production plateaued (e.g. after 60 min). We consequently sampled the reaction mixture containing methylglyoxal-adducted NacCys and the various DJ-1 mutants at 15 min and 60 min for the presence of lactate. Again, the four pathogenic mutations (A39S, E64D, E163K, and A179T) had normal enzymatic activity, whereas three pathogenic mutations (M26I, A104T, and D149A) reduced activity, and two pathogenic mutations (L10P and L166P) completely inhibited DJ-1 activity, as did mutations (E18A and C106S) of the catalytic site (Fig. 6C). The loss of enzymatic activity in the L10P and L166P mutants might be attributable to the fact that they do not form dimers^49^.

### Wild type and mutant DJ-1 proteins purified from mammalian cultured cells exhibit similar enzymatic characteristics

**Figure 7:**
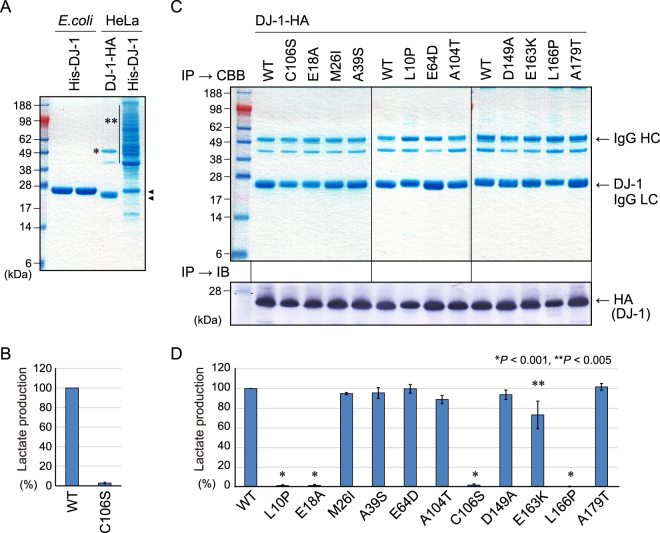
(**A**) CBB stained gels of recombinant DJ-1 proteins. His_6_-tagged WT DJ-1 (lane 1) and the C106S mutant (lane 2) purified from *E. coli*, HA-tagged WT DJ-1 immunoprecipitated from HeLa cells (lane 3), and His_6_-tagged WT DJ-1 purified from HeLa cells (lane 4). Arrowheads show the positions of the His_6_-tagged and HA-tagged DJ-1. A single asterisk indicates the IgG heavy chain and double asterisks indicate contaminating proteins. (**B**) Lactate production of HeLa cell-derived immunoprecipitated WT DJ-1 when incubated with NAC and methylglyoxal. The C106S mutation abolished lactate production. (**C**) Immunoprecipitation products of various DJ-1 mutants were subjected to CBB staining to confirm protein purity and immunoblotted for quantification. Mutant proteins were immunoprecipitated in three steps with WT DJ-1 included as a standard in each purification step. (**D**) Lactate production of various DJ-1 mutants immunoprecipitated from HeLa cells. Bars represent the mean ± SD (error bars) with statistical significance evaluated using one-way-ANOVA and Dunnett’s test.

Finally, we examined whether pathogenic DJ-1 proteins purified from mammalian cultured cells also negatively affected enzymatic activity. We initially tried to purify amino-terminal His-tagged DJ-1 from HeLa cells using nickel-agarose similar to that done in the *E. coli* expression study (Fig. 7A, lanes 1, 2). However, many non-specific proteins contaminated the eluted fraction (Fig. 7A, lane 4), we thus used immunoprecipitation to purify HA-tagged DJ-1 proteins from HeLa cells. CBB staining confirmed sufficient purity of the immunoprecipitated HA-tagged DJ-1 protein (Fig. 7A, lane 3). When HA-tagged WT and C106S DJ-1 were immunoprecipitated from HeLa cells and reacted with hemithioacetal [CH3COCH(OH)-S-NacCys], WT DJ-1 generated L-lactate, whereas the C106S mutant did not (Fig. 7B). The nine pathogenic mutants and two catalytic mutants were similarly immunoprecipitated and their purities confirmed by CBB staining (Fig. 7C, upper panel). Because the immunoprecipitates retained mouse IgG heavy/light chains that disturb stringent quantification by conventional methods, the products were immunoblotted using a rat-derived primary antibody and a secondary anti-rat IgG antibody that does not react with mouse IgG (Fig. 7C, see Methods), and the band intensity for each mutant was quantified. We then monitored the lactate production activities of the purified proteins as in Fig. 6C and normalized the results to the respective protein levels. We confirmed that even when purified from cultured mammalian cells, DJ-1 enzymatic activity is negatively affected by the pathogenic mutations L10P, E163K, and L166P (Fig. 7D). Taken together, the data shown in Figs 5, 6 and 7 demonstrate that detoxification of methylglyoxal-adducted thiols is impaired (M26I, A104T, and D149A when purified from *E. coli* and E163K purified from HeLa cells) or abolished (L10P and L166P when purified from both *E. coli* and HeLa cells) by pathogenic mutations, suggesting the pathological significance of disrupted DJ-1 activity.

## Discussion

Recent studies of PINK1 and Parkin have shed light on their molecular function, which has enabled us to deduce how their dysfunction causes *PINK1*- and *PARKIN*-associated familial Parkinson’s disease (reviewed in^50–57^). This progress is juxtaposed with that of DJ-1, which was identified thirteen years ago as a causative gene for familial Parkinson’s disease^1^, and yet we are still struggling to determine how dysfunction of DJ-1 triggers the disease. Among the obstacles preventing a clear understanding of DJ-1 are the diverse functions attributed to the protein by numerous published reports, some of which contradict one another. As a result, a consensus on the molecular function of DJ-1 has yet to be reached. Uncovering the genuine function of DJ-1 has thus been a goal of the community for sometime. We show in this paper that DJ-1 functions in thiol quality control by converting thiols such as CoA or its intermediate (β-alanine) from a methylglyoxal-adduct. The simplest interpretation is that DJ-1 directly converts methylglyoxal-conjugated low-molecular thiol (such as GSH and CoA) to intact thiol and L-lactate. However, because hemithioacetal formation is a reversible reaction, it is possible that DJ-1 accelerates the counter reaction by methylglyoxal consumption and consequently restores the methylglyoxal-conjugated thiol. Although we cannot rule out this possibility, DJ-1 converted the methylglyoxal-adducted CoA more efficiently than methylglyoxal alone (Fig. 3F), suggesting DJ-1 directly functions on methylglyoxal-adducted CoA. In both cases, it is important to note that unlike glyoxalase I, DJ-1 can convert methylglyoxal-conjugated CoA and β-alanine to intact molecules (Figs 3 and 4, see later Discussion).

A unique characteristic of DJ-1 that distinguishes it from other Parkinson’s disease-relevant proteins, such as PINK1 and Parkin, is its evolutionary conservation with the prokaryotic kingdom. The *E. coli* protein YajL has 38% amino acid identity and 50% similarity with human DJ-1, and a comparison of their crystal structures revealed that YajL and DJ-1 have almost identical backbone structures with an Cα RMSD value of 2.01 Å (Fig. 2C,D). Such a high similarity in both sequence and structure suggests that human DJ-1 and its prokaryotic homolog have highly related biochemical activities. Analysis of operons that include the prokaryotic DJ-1 homolog revealed that the *B. subtilis* DJ-1 homolog, *YraA*, comprises an operon with formaldehyde dehydrogenase *AdhA* ^31^, suggesting a potential role in aldehyde detoxification. The *E. coli* counterpart of DJ-1, *YajL*, forms part of an operon with *PanE*, implying participation in the CoA biosynthetic pathway (Fig. 2E). The DJ-1 family protein, Hsp31, and DJ-1 itself have been reported to have glyoxalase III activity^32–34^, indicating a role in methylglyoxal detoxification.

Under physiological conditions, methylglyoxal spontaneously reacts with the thiol on intracellular glutathione (GSH), and methylglyoxal-conjugated GSH is degraded by the glyoxalase I/II cascade^35,36^. When considering intracellular thiols, GSH is the most abundant thiol in cells and has protective roles against reactive oxygen, nitrogen species, and electrophiles^58^. However, CoA is also a critical thiol *in vivo*. Based on the totality of this information, we inferred that DJ-1 restores methyglyoxal-attacked CoA and a CoA intermediate, β-alanine. We confirmed that DJ-1 converts methylglyoxal-adducted CoA or β-alanine to intact CoA or β-alanine with L-lactate (Figs 3 and 4). The classical methylglyoxal detoxification system (glyoxalase I/II system) has been reported to only convert methylglyoxal-conjugated GSH, a specificity that we confirmed by showing that yeast glyoxalase I is unable to degrade methylglyoxal-conjugated CoA (Fig. 3F). When intracellular GSH is heavily oxidized or sequestered with other toxic reagents, GSH cannot interact with intracellular methylglyoxal. Under these conditions, the glyoxalase I/II system cannot effectively counteract methylglyoxal reactions with various intracellular compounds including CoA and β-alanine. DJ-1, however, offers a viable cellular alternative to dealing with methylglyoxal-conjugated compounds.

Several pathogenic mutations impair or abolish the enzymatic activity of DJ-1 when purified from *E. coli* or mammalian cells (Figs 5 to 7), suggesting physiological relevancy. Intriguingly, the enzymatic activity of the DJ-1 E163K mutation was affected when purified from HeLa cells but not when purified from *E. coli*. We recently found that the subcellular localization of this mutant differs from WT DJ-1^59^, this altered localization might contribute to the different enzymatic activity observed when purified from HeLa cells. We speculate that pathogenic DJ-1 mutants with normal enzymatic activities such as A39S, E64D, and A179T are also negatively affected via some other defect such as oxidation under physiological conditions^60^.

Recently, the biosynthesis of CoA has attracted renewed attention because this pathway is involved in hereditary forms of neurodegenerative diseases. Two human enzymes involved in CoA biosynthesis, PANK2 (human homolog of PanK/CoaA, Fig. 2E) and COASY (human homolog of CoaD and CoaE, Fig. 2E), are associated with a hereditary disease characterized by neurodegeneration with brain iron accumulation (NBIA)^61,62^. Of note, one of the clinical symptoms of NBIA is Parkinsonism^63,64^, suggesting a link between CoA biosynthesis and Parkinsonism. Moreover, CoA is an obligatory cofactor for mitochondrial function. The CoA thiol reacts with acyl groups to make thioester derivatives, such as acetyl-CoA and succinyl-CoA, both of which are essential for the citric acid cycle (also known as the TCA cycle or the Krebs cycle) to supply NADH for the mitochondrial respiratory chain^42^. It is thus reasonable that dysfunction of DJ-1 in CoA quality control against aldehyde attack causes mitochondrial abnormality *in vivo* ^18,21,22,25,26^. It is also reasonable that dysfunction of DJ-1 causes Parkinsonism as dysfunction of genes involved in the CoA biosynthetic pathway (*PANK2* and *COASY*) trigger neurodegeneration with Parkinsonism. Because data are limited to *in vitro* biochemical analysis, further cellular analyses are required. However, we believe that our hypothesis of the genuine DJ-1 function - an aldehyde-adduct hydrolase that protects low-molecular-weight thiols, including CoA, from attack by methylglyoxal - is a solid springboard for further studies.

## Methods

### Analysis of prokaryotic DJ-1 homologs

Prokaryotic homologs of DJ-1 were identified using Basic Local Alignment Search Tool (BLAST, http://blast.ncbi.nlm.nih.gov/Blast.cgi). The prokaryotic operon structure was examined using the ODB3 (http://operondb.jp/)^44,45^ and DOOR (http://csbl.bmb.uga.edu/DOOR/)^46,47^ databases. To compare the human DJ-1 structure with that of the prokaryotic homologs, structural coordinates of human DJ-1 (PDB accession code 1P5F), *E. coli* Hsp31/HchA (PDB accession code 1IZY), and *E. coli* YajL (PDB accession code 2AB0) were superimposed using Secondary Structure Matching (SSM) in CCP4.

### Preparation of DJ-1 proteins

To obtain recombinant DJ-1 proteins from *E. coli*, WT and mutant *DJ-1* genes were subcloned into pET21a(+) and pET28a(+) plasmids (Novagen - Merck Millipore), and then transformed into the *E. coli* BL21(DE3) + RIL strain (Agilent Technologies). His_6_-tagged WT DJ-1 and various DJ-1 mutants were purified by standard procedures using nickel-agarose (Ni-NTA Agarose, Qiagen) and elution buffer [200 mM NaCl, 10 mM 2-mercaptoethanol, and 500–750 mM imidazole in 20 mM sodium phosphate buffer (pH7.0)]. Recombinant DJ-1 proteins were dialyzed using 20 mM sodium phosphate buffer (pH7.0) plus 200 mM NaCl, and stored at −80 °C. The WT DJ-1 and associated mutants were subjected to SDS-PAGE followed by CBB staining to confirm that the purified proteins resolved as single bands.

WT DJ-1 and associated mutants were also purified from cultured mammalian cells by immunoprecipitation. To prepare plasmids to express DJ-1-HA in cultured cells, wild type DJ-1 was first inserted into pcDNA3 (Invitrogen - Thermo Fisher Scientific) with a carboxyl terminal HA-tag, and various DJ-1 mutants were constructed by conventional site-directed mutagenesis^59^. *DJ-1* knock out HeLa cells made previously^59^ were transfected using the transfection reagent FuGene6 (Promega and Roche), and cells were cultured at 37 °C with 5% CO_2_ in Dulbecco’s Modified Eagle Mediums (DMEM, Sigma-Aldrich) supplemented with 10% fetal bovine serum (Equitech-BIO), 1 × penicillin-streptomycin-glutamine (Life Technologies), 1 × non-essential amino acids (Life technologies), and 1 × sodium pyruvate (Life Technologies). Cells were lysed with TNE-N + buffer [20 mM Tris-HCl pH8.0, 150 mM NaCl, 1 mM EDTA, 1% NP-40, and protease-inhibitor cocktail (Roche)] and lysates were centrifuged at 16,500 × g for 10 min. The HA-tagged proteins were purified from the supernatant by immunoprecipitation with anti-HA antibody conjugated agarose (A2095, Sigma-Aldrich), and enzymatic activities were examined. The purity of all immunoprecipitated DJ-1 proteins was confirmed by SDS-PAGE followed by CBB staining (Fig. 6C). Furthermore, to facilitate protein quantification, immunoprecipitated samples were immunoblotted and DJ-1 signal intensities calculated in Image J (https://imagej.nih.gov/ij/). To avoid cross-reaction with mouse IgG heavy/light chains in the anti-HA antibody conjugated agarose immunoprecipitated products, a rat anti-HA antibody (clone 3F10, Roche, 1:1,000) and a goat anti-rat IgG antibody (Sigma-Aldrich) were used in immunoblotting.

### Monitoring DJ-1 enzymatic activity

To determine if DJ-1 consumes hemithioacetals [CH_3_COCH(OH)-S-thiols] such as methylglyoxal-adducted glutathione (GSH), CoA, or n-acetyl-cysteine, 7.5 mM methylglyoxal (Sigma-Aldrich) and 7.5 mM L-GSH (Sigma-Aldrich), 7.5 mM CoA (Wako and Oriental Yeast), or 7.5 mM n-acetyl-cysteine (Sigma-Aldrich) were pre-incubated for 30 min at room temperature in 50 mM sodium phosphate buffer (pH 7.0). Then 20 µM WT DJ-1 or various DJ-1 mutants were added to the reaction mixture. Changes in hemithioacetal levels were monitored as a time-dependent change in the characteristic absorbance at 288 nm (Abs_288_) on an Enspire plate reader (Perkin Elmer). Yeast glyoxalase I (Sigma-Aldrich) activity was similarly monitored with clear evidence for methylglyoxal-adducted GSH degradation observed (not shown); in contrast, no degradation was seen with methylglyoxal-adducted CoA.

To assess DJ-1 conversion of β-alanine-, GSH-, CoA- or n-acetyl-cysteine-conjugated methylglyoxal to lactate, the production of L- or D-lactate was monitored. Methylglyoxal and GSH, CoA, or n-acetyl-cysteine (all 7.5 mM) were incubated in 50 mM sodium phosphate buffer (pH 7.0) for 30 min at room temperature prior to the start of the assay, then 20 µM purified WT DJ-1 or various DJ-1 mutants were added to the reaction mixture (t = 0). For β-alanine reactions, a higher concentration of methylglyoxal was used as described by Richarme *et al*.^37^. The 80 mM methylglyoxal and 80 mM β-alanine were incubated in 50 mM sodium phosphate buffer (pH 7.0) for 40 min at room temperature prior to the start of the assay, diluted 10 fold, and then 20 µM purified WT DJ-1 or various DJ-1 mutants were added (t = 0). DJ-1-containing reaction mixtures (20 µl) were sampled at 15 min and 60 min, and mixed with Reaction buffer II, which was prepared by mixing 250 µl glycine-hydrazine buffer (pH 9.2) (G5418, Sigma-Aldrich), 25 µl of 17 mg/ml β-NAD^+^ solution (Oriental Yeast), and 2 µl LDH enzymes [L-LDH (Oriental Yeast) for L-lactate monitoring or D-LDH (Oriental Yeast) for D-lactate monitoring]. Lactate production was detected indirectly by coupling to NADH production, which was monitored by Abs_340_ on a spectrophotometer (Nano-drop, Thermo Scientific). To confirm the fidelity of the assay, L-lactic acid (Wako) and D-lactic acid (Tokyo Chemical Industry) were used as positive controls. All assays were performed in three independent experiments with results analyzed using one-way-ANOVA and group comparisons done using Dunnett’s test.

### HPLC analysis

For measurement of intact β-alanine or methylglyoxal-conjugated β-alanine, a Shimadzu HPLC system equipped with a Triart Diol-HILIC column (250 × 4.6 mm, YMC) was used. A flow rate of 1 ml/min was used with an 80:20 mix of acetonitrile:10 mM ammonium acetate as the mobile phase. Signals were detected at 210 nm at 40 °C and the retention times of β-alanine and methylglyoxal-conjugated β-alanine were defined as ~ 13 min and 14.5 min, respectively. For measurement of intact GSH or methylglyoxal-conjugated GSH, a Shimadzu HPLC system equipped with an Inertsil ODS-3 column (250 × 4.6 mm, YMC) was used. A flow rate of 1 ml/min was used with a 5:95 mix of acetonitrile:20 mM H3PO4 + 10 mM NaClO as the mobile phase. Signals were detected at 200 and 210 nm at 40 °C and the retention times of GSH and methylglyoxal-conjugated GSH defined as ~4.5 min and 5.5 min, respectively.

### NMR analysis

NMR experiments were performed at 303 K and 298 K on an AVANCE III 600 instrument equipped with a TCI CRYOPROBE (Bruker). NMR samples included 50 mM sodium phosphate buffer (pH 7.5) in 90% H_2_O and 10% D_2_O. The final concentrations of methylglyoxal and CoA were 25 mM or 12.5 mM for the assignments, and 7.5 mM for enzymatic reaction monitoring. Both HSQC and HMBC experiments incorporated gradient coherence selection. All NMR spectra were processed and analyzed with Topspin 3.2 (Bruker). Chemical shifts were indirectly referenced using 2,2-dimethyl-2-silapentane-5-sulfonic acid (DSS).

### Data availability statement

The authors declare that all materials, data and associated protocols are available to readers with due qualifications in material transfer agreements.

## Electronic supplementary material

Supplementary Information
