# Supplementary material for: Parkinson’s disease-related DJ-1 functions in thiol quality control against aldehyde attack in vitro
Source: Sci Rep. 2017 Oct 9;7:12816. doi: 10.1038/s41598-017-13146-0 (PMC5634459; doi:10.1038/s41598-017-13146-0)

Supplementary File

Parkinson's disease-related DJ-1 functions in thiol quality control against aldehyde attack in vitro

Noriyuki Matsuda[1, 2], Mayumi Kimura[1, 3], Bruno Barros Queliconi[1, 3], Waka Kojima[1, 3, 4], Masaki Mishima[5], Kenji Takagi[5], Fumika Koyano[1], Koji Yamano[1], Tsunehiro Mizushima[6], Yutaka Ito[5], and Keiji Tanaka[3, 4]

[1]Ubiquitin Project, Tokyo Metropolitan Institute of Medical Science, 2-1-6 Kamikitazawa, Setagaya, Tokyo 156-8506, Japan, [2]JST, PRESTO, 4-1-8 Honcho, Kawaguchi, Saitama 332-0012, Japan, [3]Laboratory of Protein Metabolism, Tokyo Metropolitan Institute of Medical Science, 2-1-6 Kamikitazawa, Setagaya, Tokyo 156-8506, Japan, [4]Department of Computational Biology and Medical Sciences, Graduate School of Frontier Sciences, The University of Tokyo, 5-1-5 Kashiwanoha, Kashiwa, Chiba 277-8561, Japan, [5]Graduate School of Science and Engineering, Tokyo Metropolitan University, 1-1 Minamiosawa, Hachioji, 192-0397, Japan, [6]Picobiology Institute, Graduate School of Life Science, University of Hyogo, 3-2-1 Kouto, Kamighori, Ako, Hyogo 678-1297, Japan.

Online supplemental material

Supplementary Figure 1 (Fig. S1)

CBB staining data of WT DJ-1 and mutants purified from *E. coli,* indicating the purity of the recombinant DJ-1 proteins used in this study. Red asterisks show the fraction used.

Supplementary Figure 2 (Fig. S2)

Superimposition of heteronuclear single-quantum correlation (HSQC) spectra (red) and heteronuclear multiple bond correlation (HMBC) spectra (blue) to trace the connectivities among K', J', and L1.

Supplementary Figure 3 (Fig. S3)

The L1 peak in Fig. 3D was not reduced following incubation in the absence of DJ-1.

Supplementary Figure 4 (Fig. S4)

A lactate-derived signal was observed following incubation with DJ-1. (A) Possible molecular species present in the reaction. A - M indicate positions of the chemical shift assignments. (B) HSQC spectra of reacted methylglyoxal and CoA followed by incubation with DJ-1. Increases in M4 (derived from the CH3 moiety of lactate) and L4 (derived from the C-H moiety of lactate) were observed. A reduction in the K' peak and an increase in the K peak were also observed because methylglyoxal-conjugated CoA containing K' is converted to intact CoA containing K. Similarly, a reduction in the J' peak and an increase in the J peak should be observed, however, these two peaks

are too close to resolve by 1D-HSQC. Reductions in the CH3 moiety-derived signals (M1-M3) of free and reacted methylglyoxal were also observed because they are converted to lactate.

Online supplemental material is available at http://www.nature.com/articles/srepXXXX.

Supplementary Fig. 1

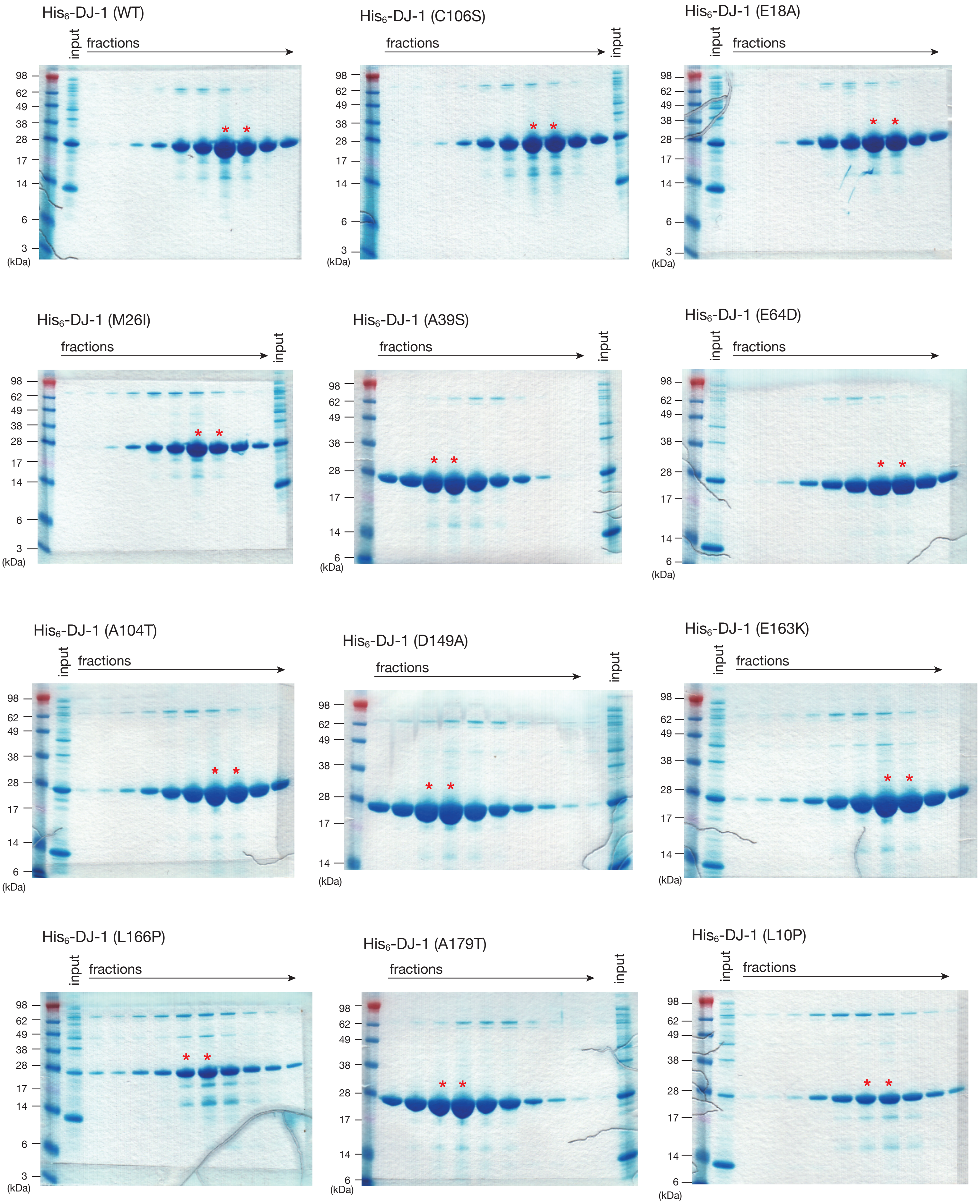

Supplementary Fig. 2

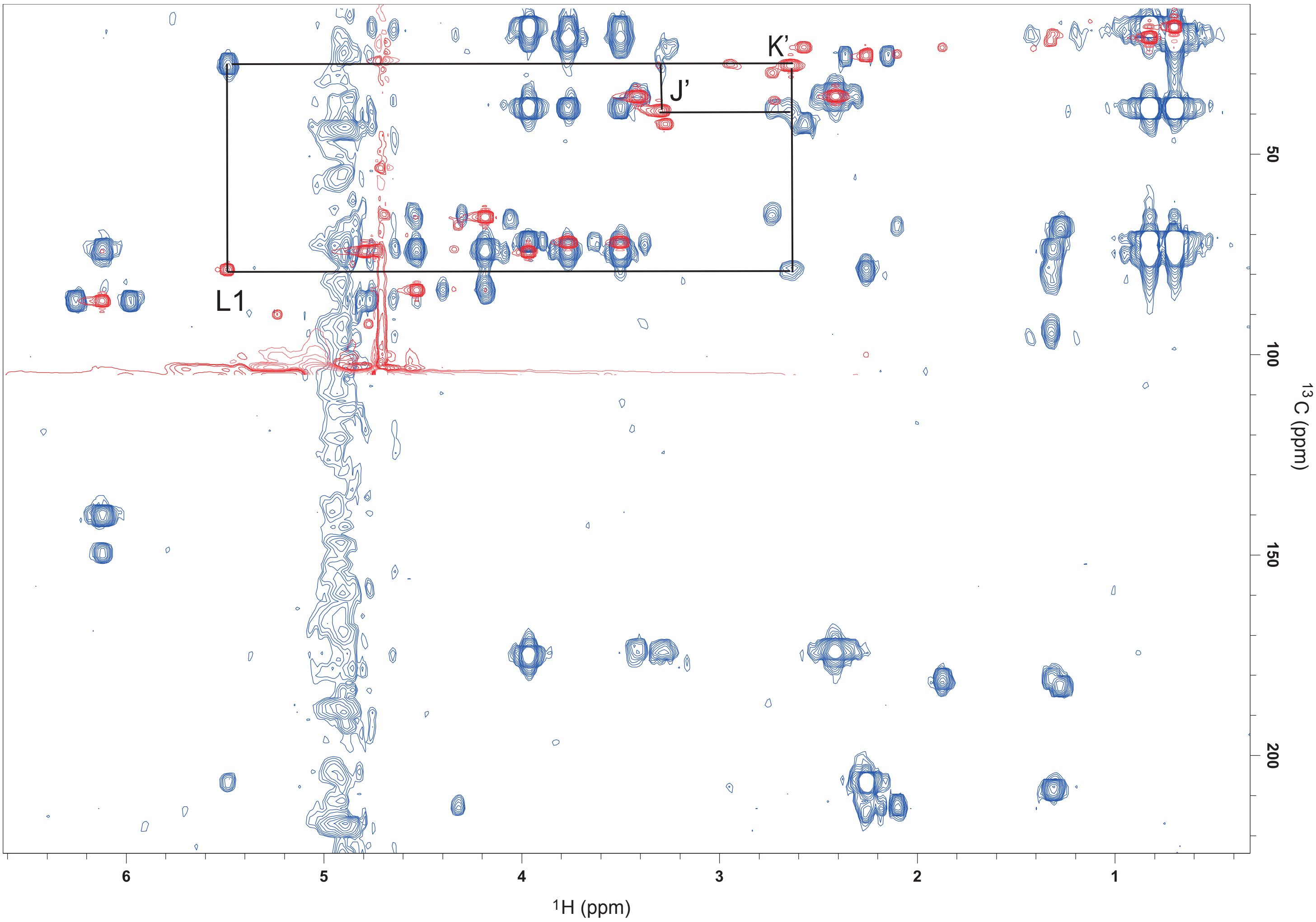

Supplementary Fig. 3

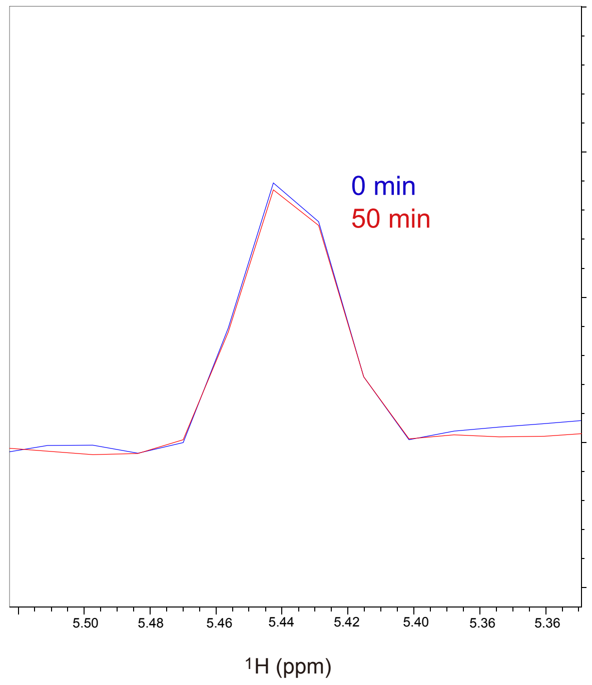

Supplementary Fig. 4

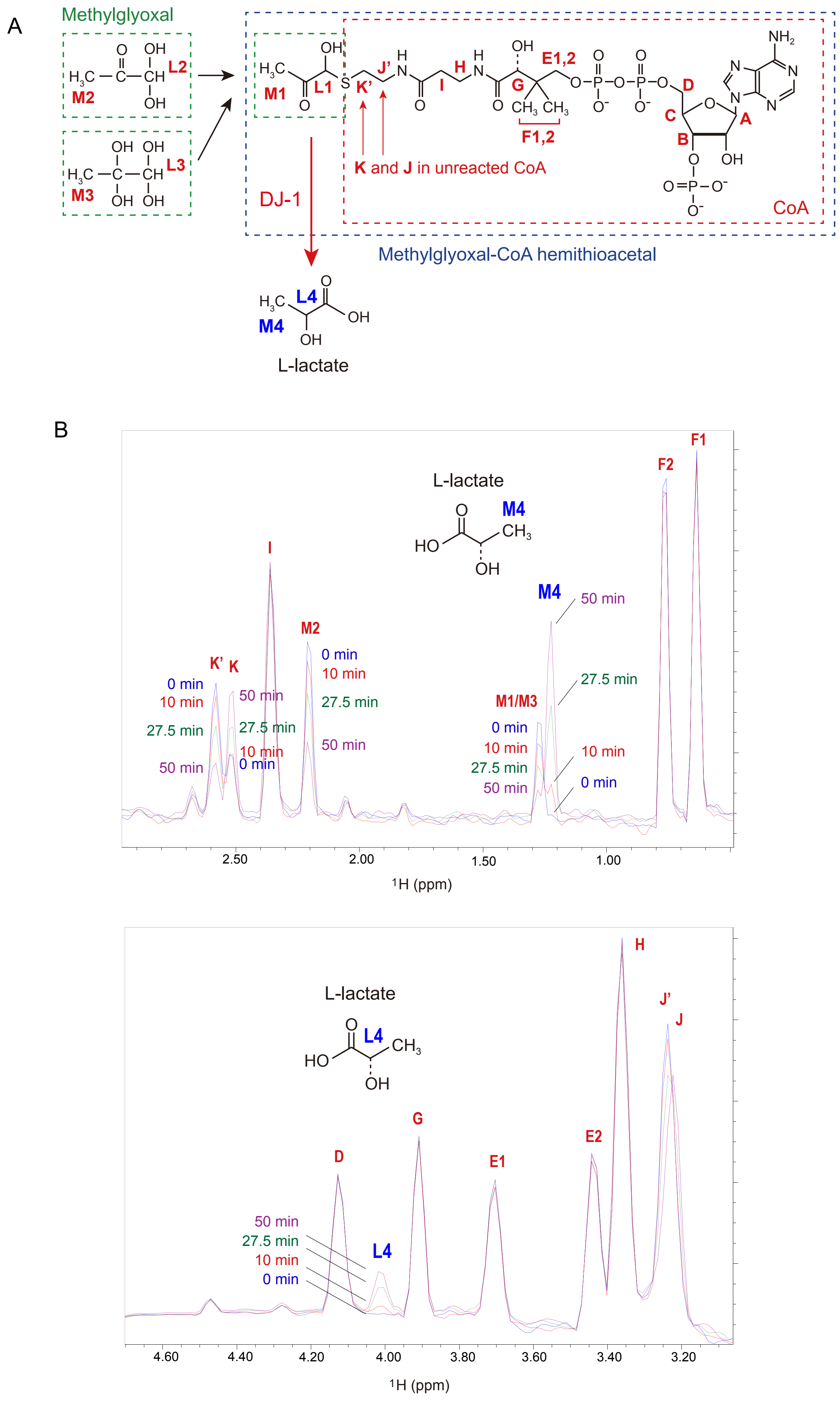

Supplement: Supplementary file 1 — Supplementary Information [file 41598_2017_13146_MOESM1_ESM.pdf]
